# Draft Genome Sequence of the Extremely Halophilic Bacterium Halomonas salina Strain CIFRI1, Isolated from the East Coast of India

**DOI:** 10.1128/genomeA.01321-14

**Published:** 2015-01-08

**Authors:** Bijay Kumar Behera, Priyanka Das, Jitendra Maharana, Prasenjit Paria, Shambhu Nath Mandal, Dharmendra Kumar Meena, Anil Prakash Sharma, Rijith Jayarajan, Vishal Dixit, Ankit Verma, Shamsudheen Karuthedath Vellarikkal, Vinod Scaria, Sridhar Sivasubbu, Atmakuri Ramakrishna Rao, Trilochan Mohapatra

**Affiliations:** a Biotechnology Laboratory, Institute for Conflict Analysis and Resolution, Central Inland Fisheries Research Institute, Kolkata, India; b Institute for Conflict Analysis and Resolution, Central Inland Fisheries Research Institute, Kolkata, India; c Genomics and Molecular Medicine Unit, CSIR-Institute of Genomics and Integrative Biology, New Delhi, India; d GN Ramachandran Knowledge Center for Genome Informatics, CSIR-Institute of Genomics and Integrative Biology, New Delhi, India; e Academy of Scientific and Innovative Research, New Delhi, India; f Centre for Agricultural Bioinformatics, Institute for Conflict Analysis and Resolution, Indian Agricultural Statistics Research Institute, New Delhi, India; g Institute for Conflict Analysis and Resolution, Central Rice Research Institute, Cuttack, India

## Abstract

Halomonas salina strain CIFRI1 is an extremely salt-stress-tolerant bacterium isolated from the salt crystals of the east coast of India. Here we report the annotated 3.45-Mb draft genome sequence of strain CIFRI1 having 86 contigs with 3,139 protein coding loci, including 62 RNA genes.

## GENOME ANNOUNCEMENT

Halophilic and halotolerant bacteria are designated an elite class of bacteria because of their inherent capability to cope in hypersaline environmental niches. A diverse taxa of halophilic and halotolerant bacteria have been recovered from a wide variety of hypersaline environments (1–6). The isolation and characterization of halophilic and halotolerant bacteria from hypersaline environments could provide interesting insights into their evolutionary and survival mechanisms, including the study of halotolerant genes (7, 8). These organisms have also been studied with respect to their use in production of useful biomolecules, such as osmolytes (compatible solutes), hydrolytic enzymes, and exopolysaccharides (9). Halomonas salina strain CIFRI1 is an extremely salt-stress-tolerant bacterium isolated from salt crystals in this study. Herein, we report the draft genome sequence of Halomonas salina strain CIFRI1 for the first time.

Halomonas salina strain CIFRI1, an extremely halophilic bacteria, was isolated from salt crystals collected from the salt pan at Digha in West Bengal, along the east coast of India (21°37′36.51″N 87°31′20.23″E). The strain was isolated using liquid tryptic soya broth (TSB) medium (10) along with a final concentration of 2% NaCl, pH 7.3 ± 0.2, with overnight incubation at 37°C. The cultures were further replated with tryptic soy agar (TSA) medium (11) with incubation for 2 to 3 days. Based on the differences in the colony morphology and Gram-staining reaction, an individual bacterial colony was selected for serial dilution. Bacteria that grew significantly in the presence of >25% NaCl in TSB medium during overnight incubation at 37°C were selected for molecular characterization and whole-genome sequencing. Genomic DNA was extracted using the Mobio Soil DNA isolation kit (Mobio, USA) according to the manufacturer’s instructions, and 16S rRNA gene sequencing was done using universal bacterial primers (12) along with BigDye Terminator cycle sequencing (Applied Biosystems, USA). The 16S rRNA gene sequence of CIFRI1 is closely related to that of Halomonas salina based on the biochemical and BLAST results. The BLAST result showed 99.65% homology with the Halomonas salina strain F8- 11 16S rRNA gene (NR_042050).

The genome annotations were performed by the NCBI Prokaryotic Genomes Annotation Pipeline (13). A total of 1,356,989 paired-end reads with a read length of 101 bp were generated by using HiSeq 2500 (Illumina, USA) according to protocols supplied by the manufacturer. Adapter-trimmed high-quality reads were used for the assembly using CLC Genomic Workbench version 7.0.3 *de novo* assembler (Qiagen, NL). The primary assembly consisted of 86 contigs (*N*_50_ = 68,789), and further scaffolding resulted in 67 scaffolds with an *N*_50_ value of 493,653 bases. This draft genome of Halomonas salina had a total of 3,450,272 bases. Furthermore, the automated gene annotation by RAST (14) revealed 3,139 protein-coding loci including 62 RNA genes.

### Nucleotide sequence accession numbers.

The whole-genome shotgun project has been deposited at DDBJ/EMBL/GenBank under the accession number JOKD00000000. The version described in this paper is JOKD00000000.1.
