# Erratum for Behera et al., Draft Genome Sequence of the Extremely Halophilic Bacterium *Halomonas salina* Strain CIFRI1, Isolated from the East Coast of India

**DOI:** 10.1128/genomeA.00123-15

**Published:** 2015-02-19

**Authors:** Bijay Kumar Behera, Priyanka Das, Jitendra Maharana, Prasenjit Paria, Shambhu Nath Mandal, Dharmendra Kumar Meena, Anil Prakash Sharma, Rijith Jayarajan, Vishal Dixit, Ankit Verma, Shamsudheen Karuthedath Vellarikkal, Vinod Scaria, Sridhar Sivasubbu, Atmakuri Ramakrishna Rao, Trilochan Mohapatra

**Affiliations:** a Biotechnology Laboratory, ICAR-Central Inland Fisheries Research Institute, Kolkata, India; b ICAR-Central Inland Fisheries Research Institute, Kolkata, India; c Genomics and Molecular Medicine Unit, CSIR-Institute of Genomics and Integrative Biology, New Delhi, India; d G. N. Ramachandran Knowledge Center for Genome Informatics, CSIR-Institute of Genomics and Integrative Biology, New Delhi, India; e Academy of Scientific and Innovative Research, New Delhi, India; f Centre for Agricultural Bioinformatics, ICAR-Indian Agricultural Statistics Research Institute, New Delhi, India; g ICAR-Central Rice Research Institute, Cuttack, India

## ERRATUM

Volume 3, no. 1, e01321-14, 2015. Page 1: The affiliation line should read as given above.

